# Reliable Correlational Cuing While Controlling for Most-Recent-Pairing Effects

**DOI:** 10.3389/fpsyg.2020.592377

**Published:** 2020-11-16

**Authors:** Guangjun Xu, J. Toby Mordkoff

**Affiliations:** Department of Psychological and Brain Sciences, University of Iowa, Iowa City, IA, United States

**Keywords:** correlational cuing, contingency learning, stimulus-response binding, associative learning, contingencies

## Abstract

Irrelevant aspects of the environment or irrelevant attributes of task-relevant stimuli can have important and reliable effects on behavior. When the specific values of an irrelevant attribute are correlated with different responses, a correlational-cuing effect is observed: faster and more accurate responses when the correlation is positive. Previous work has shown that this effect is not due to simple differences in how often the specific stimuli or attributes are being presented, and most explanations of the effect have stressed the clear parallels with classical associative learning. There are alternative explanations, however, that center on instances, episodes, or events, instead of associative learning. One such model posits that transient bindings between irrelevant stimulus attributes and responses (i.e., most-recent-pairings) may be responsible for the correlation-cuing effect and some recent work has found no evidence of correlational cuing when most-recent-pairings are taken into account. However, the experimental conditions that were employed previously may not have been optimized for associative learning. A new experiment that was designed to emphasize associative learning was conducted and produced reliable evidence of correlational cuing even when controlling for most-recent-pairing effects.

## Introduction

Events in the world are not random. Certain objects appear most often in certain contexts and certain behaviors or responses are more likely in certain situations. The mind is sensitive to these facts and the study of the mechanisms that perform the required processing has always been central to experimental psychology, from early work on classical conditioning (e.g., Pavlov, 1927; Rescorla and Wagner, 1972) to recent work on selective attention (e.g., Mordkoff and Halterman, 2008) and cognitive control (e.g., Schmidt and Besner, 2008). In the literature, these relationships have been referred to by various labels, from *internal constraints* (e.g., Garner, 1962) to [biased] *contingencies* (e.g., Miller, 1987; Mordkoff and Yantis, 1991; Schmidt and De Houwer, 2012). The present work concerns a specific type of contingency effect, known as *correlational cuing*, and the possible causal mechanisms.

Because it involves an irrelevant stimulus or irrelevant attribute of a target stimulus, correlational cuing is similar to the Stroop Effect (Jaensch, 1929; Stroop, 1935), Simon Effect (Simon and Rudell, 1967), and Flanker Effect (Eriksen and Eriksen, 1974). The key difference is that correlational cuing does not rely upon any conceptual or instruction-based overlap between the irrelevant attribute and the response. Instead, correlational cuing is due to certain mathematical relationships that are included within the experimental design, which are thought to create associations between specific values of the irrelevant stimulus attribute and one or more of the available responses.

As an example, consider the experimental design that is shown in Table 1 (which is based on the first published experiment concerning correlational cuing; Miller, 1987, Expt. 1). On each trial, a single colored letter is shown and the instructions require one of two responses, depending on the color, with two colors assigned to each response. The participants are told that the shape of the stimulus is irrelevant, but, as can be seen, each of the two letters is strongly correlated with one of the responses. In the example provided by Table 1, the irrelevant attribute ***X*** is strongly associated with the left-hand response, because 88 of the 96 trials that involve the letter ***X*** require a left-hand response. The opposite holds for the letter ***O***. When experiments like this are conducted (e.g., Miller, 1987; Schmidt et al., 2007; Cosman et al., 2016; Giesen et al., 2020; Schmidt et al., 2020), one observes a *correlational-cuing effect*: an advantage for trials on which the irrelevant attribute is positively correlated with the correct response.

**TABLE 1:** First example correlational cuing design.

Relevant attribute	Correct response	Irrelevant attribute	
		*X*	*O*
Red	Left	44	4
Green	Left	44	4
Blue	Right	4	44
Yellow	Right	4	44

**The numbers are trials per block (of 192 trials) with the particular combination of (relevant) color and (irrelevant) letter.**

### Display-Frequency Effects

The original explanation of correlational cuing is that participants, over the course of many trials, learn the relationship between the irrelevant attribute and the correct response (e.g., ***X*** *→ left* and ***O*** *→ right*; see, e.g., Miller, 1987; Mordkoff, 1996; Danek, 2010; Schmidt and De Houwer, 2016a,b, 2019). However, there is an alternative explanation, at least when the design is similar to that shown in Table 1 (for more discussion, see Miller, 1987; Schmidt et al., 2007). This alternative focuses on the frequencies of specific displays, instead of the correlations between irrelevant attributes and available responses. In the design shown in Table 1, for example, each of the specific displays used for the positive correlation condition occurs 11 times as often as each of the displays for negative correlation (44 vs. 4). This difference in display frequency could cause the effect without any learning of the relationships between irrelevant attributes and correct responses. First, displays that are presented more often could be encoded and processed more quickly (e.g., Irwin and Pachella, 1985). Second, displays that are presented more often are more likely to be preceded by the exact same display, which has long been known to confer a performance advantage (e.g., Bertelson, 1961; Pashler and Baylis, 1991).

To address this alternative, a more-complicated design has been used (see Miller, 1987; Expt. 2; Schmidt et al., 2007, Expt. 4). One such design is shown in Table 2. Note, first, the correlation between values of the irrelevant attribute and the correct response: for example, 76 of the 96 trials involving the letter ***X*** require a left-hand response. Next, note how this relationship is created by the “inducing trials” that occur at unequal frequencies (30 vs. 2). In contrast, the four cells of the table that depict “test trials” all occur at the same frequency (16 per block), but still include the same correlation between the irrelevant attribute and correct response. That the correlational-cuing effect is just as large for test trials as it is for inducing trials (Miller, 1987; Schmidt et al., 2007) would appear to rule out the alternative explanation that focuses entirely on display frequency.

**TABLE 2:** Second example correlational cuing design.

Relevant attribute	Correct response	Irrelevant attribute		Condition label
		*X*	*O*	
Red	Left	30	2	Inducing
Green	Left	30	2	Inducing
Yellow	Left	**16 (+)**	***16* (−)**	Test
Blue	Right	***16* (−)**	**16 (+)**	Test
Orange	Right	2	30	Inducing
Purple	Right	2	30	Inducing

**The numbers are trials per block (of 192 trials) with the particular combination of (relevant) color and (irrelevant) letter; cells with upright bold frequencies and (+) are positive test trials; cells in bold italics with (−) are negative trials.**

### Retrieval and Binding Effects

There is, however, another alternative to the idea that correlational cuing is due to learned associations between irrelevant attributes and available responses. This alternative includes a large family of specific models, unified by their focus on previous instances, events, and/or episodes, instead of on correlations (see Giesen et al., 2020; Schmidt et al., 2020). These instance-based models can be viewed as existing along a continuum that is defined by how many previous instances—i.e., separate experiences with the stimuli and responses that comprise the task—are allowed to have an effect on current-trial performance. At one extreme are models that allow all previous experiences to influence behavior equally; at the other extreme are models under which only one previous experience will have an effect.

An example of an instance-based model that allows multiple previous experiences to influence performance is that rooted in Parallel Episodic Processing (see Schmidt et al., 2016). According to this view (see Giesen et al., 2020; Schmidt et al., 2020), when a display attribute is encoded, many or all previous experiences involving this attribute are retrieved in parallel and the responses that were made during these previous episodes are at least partially activated. If a majority of the retrieved episodes involve a particular response, then that response will enjoy an advantage. Thus, for example, when a participant in an experiment using the design shown in Table 2 is shown the irrelevant attribute ***X***, a large majority of the retrieved episodes will involve the left-hand response, which would explain the effect that is found in performance. While critically different in terms of underlying mechanism from the associative-learning account of correlational cuing, this retrieval-based alternative is very difficult to test because it depends on the same experimental conditions (for discussions, see Giesen et al., 2020; Schmidt et al., 2020). Any change in trial frequencies that alters the correlations would have the same effect on the proportions of retrieved episodes. For this reason, the present work will not attempt to discriminate between associative learning and the retrieval of all previous episodes.

An example of an instance-based model that depends on only one previous experience is that rooted in the Theory of Event Coding (see Hommel et al., 2001). These models posit temporary “bindings” between attributes, which are constantly being created and over-written by new experiences (for a recent discussion, see Frings et al., 2020). These binding are created between all current attributes, regardless of task-relevance, including the attributes of the response. Thus, for example, when a trial with a red ***X*** results in a left-hand response, temporary links are created between ***X*** and *red*, and between ***X*** and *left hand*, even if the shape of the stimulus is task-irrelevant (as it is in the designs shown in Tables 1, 2). If trials including the letter ***X*** are more likely to require a left-hand response, then the most-recent (previous) trial involving an ***X*** is also more likely to have involved the left hand. On the idea that repeating a binding leads to better performance than changing a binding (see, e.g., Hommel, 1998, 2004; Rothermund et al., 2005; Colzato et al., 2006; Frings et al., 2007), this alternative can explain correlational cuing without reference to associative learning. Fortunately, the binding-based model is easily tested.

In contrast to the issue of display frequency reviewed earlier, one cannot simply add a new condition that avoids the effects of most-recent-pairings in order to test the binding-based model. Any manipulation that alters the long-term relationship between an irrelevant attribute and the correct response (which is what the learning-based model depends on) will also affect the probabilities of the most-recent-pairings (which is what the binding-based model depends on). Because of this, the two recent tests of instance-based models have taken a different approach and used hierarchical regression to tease them apart (Giesen et al., 2020; Schmidt et al., 2020). Each trial was categorized in terms of both long-term correlation and recent pairings. In the subsequent analyses, the question was whether either predictor could explain a significant amount of additional variance, beyond that which could be explained by the other predictor. In both of these tests, the answer was the same: while recent pairings or bindings could explain some variance that correlation condition could not, correlation condition did not explain any variance that pairings or bindings could not.

### Optimizing the Conditions for Correlation Cuing

The findings from the recent regression analyses (Giesen et al., 2020; Schmidt et al., 2020) are a serious challenge to the idea participants learn the relationships between irrelevant stimulus attributes and correct responses in a manner that matches classical learning theory—i.e., using *all* of their previous experiences combined. The results do not rule out the learning-based model of correlational cuing, but they do undermine a majority of its support. However, the specific conditions that were employed for these tests may not have been optimized for associative learning. Most of all, it could be argued that the simplicity of the employed designs—which is usually something to be preferred—may have primed the activity of recent instance-based mechanisms over those that depend on all previous experience.

Based on the state of the literature (see, e.g., Frings et al., 2020; Giesen et al., 2020; Schmidt et al., 2020), we suggest that at least three things should be done in order to optimize the conditions for associative learning over most-recent-pairing or binding effects. First, because pairing effects have been found to decrease with lag, the number of different values for the irrelevant attribute should be high (e.g., six or more), such that the average lag between appearances of the same value is high. Second, in order to shift processing toward elements that predict the response (and away from anything that focuses on stimulus values), a many-to-one mapping should be employed (see Tables 1, 2 for examples). When more than one value of the relevant attribute is assigned to each response, the critical decision is which response should be made, not which stimulus value was shown. Third, the correlation between the irrelevant attribute and correct response should be strong. A weak correlation is more likely to be “swamped” or over-shadowed by all of the possible repetition effects.

To see how previous work has not optimized the conditions for correlational cuing over most-recent-pairing effects, consider the design that is shown in Table 3 (which was used in several of the experiments that were included in the analyses of Schmidt et al., 2020). Note, first, that a one-to-one mapping was used, with each value of the relevant attribute being mapped to a different response (The same was true for the experiment of Giesen et al., 2020). Second, note that there were only three possible values for each stimulus attribute, such that repetitions across adjacent trials would be quite frequent (The experiment by Giesen et al., 2020, used four possible values). These were logical design decisions, given that the experiments were intended to mimic a simple Stroop Task using words that were not color-names, but these might not be the best conditions for the learning of associations between irrelevant attributes and specific responses. For example, when a one-to-one mapping is used, the association between the irrelevant and relevant stimulus attributes is equal to and might overshadow the association between the irrelevant stimulus attribute and correct response (see, e.g., Danek, 2010; Schmidt and De Houwer, 2016a,b, 2019). Likewise, when stimulus attributes repeat across trials very often, those mechanisms responsible for longer-term representations might be de-emphasized in favor of short-term bindings. Finally, it’s worth noting that the correlation in one previous study (Giesen et al., 2020) was quite weak (2 out of 6 when chance was 1 out of 4), which is not optimal for correlational cuing.

**TABLE 3:** Third example correlational cuing design.

Relevant attribute and correct response	Irrelevant attribute		
	*Find*	*Walk*	*Make*
Blue	8	1	1
Red	1	8	1
Green	1	1	8

**The numbers are trials per block (of 30 trials) with the particular combination of (relevant) color and (irrelevant) word.**

## Experiment

Given the importance of whether evidence of correlational cuing can be found when the effects of most-recent-pairing are removed or controlled, a new experiment was conducted using closer-to-optimal conditions. To reduce the influence of repetition effects in general and increase the lag between all repetitions, there were six different values to each stimulus attribute, instead of only three or four. To encourage a focus on the response-choice decision, a many-to-one mapping was used. The correlation between irrelevant attributes and correct responses was quite strong (5 out of 6 when chance is 1 out of 2). Finally, because the relative timing of processing may be more important to correlational cuing than to most-recent-pairing effects (see, e.g., Miller, 1986), the specific values for each stimulus dimension were selected to be approximately equal in discriminability and the assignment of stimulus dimension to role (i.e., relevant vs. irrelevant) was counter-balanced across participants.

### Participants

Thirty-two undergraduates enrolled in Elementary Psychology were each run in a single, 1-h session. The number of participants was determined by the combination of an *a priori* power analysis and the constraints imposed by counter-balancing. All participants reported “normal” or “corrected-to-normal” visual acuity, no color blindness, and no previous experience with this particular task. All provided informed consent using procedures approved by the Institutional Review Board and all were given a written or verbal explanation upon completion.

### Procedure

Each participant completed 19 blocks of trials. The first block was explicitly labeled as practice and provided complete feedback after every trial, plus a reminder display of the assigned targets-to-responses mapping after any error. For participants assigned to respond to the letter, the letters were displayed in white; for the participants responding to color, filled squares were used. The second block of practice only provided one-word (“correct” or “error”) feedback. The third practice block and all subsequent blocks only gave feedback after an error.

Each block contained 36 trials (see Table 4, below). Each trial began with the appearance of a fixation cross, which remained visible for 350 ms. After a delay of 150 ms, the target appeared at the same location and remained visible until a response was made or 2,500 ms had elapsed. The inter-trial interval following a correct response was 1,000 ms. If an error was made and feedback was given, the inter-trial interval was increased to 1,500 ms.

**TABLE 4:** Example experimental design, present experiment.

Relevant feature (Color)	Correct response	Irrelevant feature (Letter)					
		*H*	*W*	*X*	*M*	*T*	*V*
Red	Left	**1 (+)**	***1* (−)**	2	0	2	0
Orange	Left	2	0	**1 (+)**	***1* (−)**	2	0
Gold	Left	2	0	2	0	**1 (+)**	***1* (−)**
Purple	Right	0	2	0	2	***1* (−)**	**1 (+)**
Blue	Right	0	2	***1* (−)**	**1 (+)**	0	2
Green	Right	***1* (−)**	**1 (+)**	0	2	0	2

**Trial frequencies in upright bold with (+) are positive test trials; trial frequencies in bold italics with (−) are negative test trials; all other trials are inducing trials.**

At the end of each block, participants were provided with summary feedback: accuracy (in percent) and mean response time (in milliseconds). If accuracy was below 90%, the message “please slow down and be more careful” was added. The end-of-block summary was presented for a minimum of 7,500 ms.

### Stimuli, Task, and Counter-Balancing

The fixation cross was a white plus-sign, 0.32 cm square, subtending 0.28° from the standard viewing distance of 66 cm. The targets were the upper-case letters **H**, **M**, **T**, **V**, **W**, and **X**, presented in (ePrime colors) red, dark orange, gold, lime green, deep sky blue, and medium purple. Pilot testing using the flicker-fusion method suggested that these six colors are approximately equal in brightness. The target letters were presented in a bold, sans-serif font that was roughly 1.27 cm square, subtending 1.10°.

The task was two-alternative forced-choice with three targets assigned to each response. Responses were made using the left and right index fingers. Half of the participants were assigned to respond to the letter; the other half responded to color. The six letters were divided into two sets—**H**, **W**, and **X** vs. **M**, **T**, and **V**—with each set mapped to a different response. A series of pilot tests (conducted by Jacob Sherman) suggested that the six colors needed to be divided into “warm” and “cold” sets—i.e., red, orange, and gold vs. green, blue, and purple—in order to be equally difficult to classify as the two letter sets.

The same sets of letters and colors were used when the feature was task-irrelevant. Thus, there were eight counter-balancing groups, defined by which feature was task-relevant, which set of letters was assigned to or correlated with the left-hand response, and which set of colors was assigned to or correlated with the left-hand response.

### Design

Each of the six letters and six colors appeared equally often, but the 36 possible combinations of letter and color did not occur equally often, with one-third of the combinations never appearing at all (see Table 4). This imbalance creates a correlation between the irrelevant attribute and correct response. This imbalance is also what defines the three trial types. In what follows, the design will be described in terms that apply to participants who were assigned to respond to color (see Table 4); analogous designs were used for participants who were assigned to respond to the letter.

For each of three pairs of letters (e.g., ***H*** and ***W***), two of the colors assigned to each response (e.g., *orange* and *gold* for left-hand responses) only appeared as one letter (e.g., there were orange ***H***s but no orange ***W***s). These inducing trials create the correlations between these particular letters and one of the responses. The third color assigned to each response (e.g., red for left-hand responses) appeared equally often as these two letters and act as test trials. Positive test trials are defined as those involving the letter that is (positively) correlated with the correct response; negative test trials are those using the letter that is correlated with the opposite response. Note that the specific colors that are used on test trials depend on the pair of letters. When all three pairs of letters are taken into account, all six colors play a role in all three conditions.

### Data Selection and Analysis Plan

The data from the three practice blocks and the first three “real” blocks were all excluded from the analysis (as is planned, standard practice in our lab). The first three trials of each subsequent block were also excluded (as “warm-up”), as were all trials that immediately followed an error (see, e.g., Rabbitt, 1968, for rationale). For the analysis of mean response time (mRT), only correctly performed trials were included. The mRT and accuracy data were analyzed in separate but parallel, mixed-factor ANOVAs, with relevant dimension (color vs. letter) as the one between-subjects factor. The criterion for significance (i.e., risk) was 0.05. When an effect was significant, an estimate of population effect size was calculated using adjusted partial eta squared (*adj* ${\hat{\eta }}_{\text{p}}^{2}$) in order to avoid the positive bias of the unadjusted value (Mordkoff, 2019).

The plan was to conduct the analysis in three steps. The first step was designed to verify a correlational-cuing effect while ignoring the issue of most-recent-pairings (For this analysis, inducing and positive test trials were kept separate). The second step was to verify the effects of most-recent-pairing while ignoring correlation condition (For this analysis, the distinction between inducing and positive test trials is moot). The final and critical step was to re-examine the effects of correlational cuing using a sub-set of the data that was equalized in terms of most-recent-pairing effects (For this analysis, inducing and positive test trials were combined to create a single positive-correlation condition).

### Results

The first set of analyses concerned correlational cuing, ignoring the question of most-recent-pairing. In the analysis of mean RT, neither the main effect of relevant dimension (color vs. letter), nor the interaction between this factor and correlation condition was even close to reliable; both ***F*** < 1, *p* ≥ 0.482. In contrast, the main effect of correlation condition was highly reliable even after applying the Huynh-Feldt correction for a significant violation of sphericity: ***F***(1.54, 46.12) = 17.87, *p* < 0.001, *adj* ${\hat{\eta }}_{\text{p}}^{2}$ = 0.352. The mean response-times (mRTs) for inducing, positive, and negative test trials were 472.05, 467.23, and 489.19 ms, respectively. Pairwise comparisons (using Dunn-Šidák correction) revealed significant differences between positive and negative test trials (21.96 ± 4.92 ms; *p* < 0.001) and between inducing and negative test trials (17.14 ± 3.07 ms; *p* < 0.001), but not between inducing and positive test trials (4.82 ± 3.33 ms; *p* = 0.403). The mean error-rates (mERs) for inducing, positive, and negative test trials were 3.0, 2.9, and 5.3%, respectively. The main effect of correlation condition was significant: ***F***(1.79, 53.75) = 9.12, *p* < 0.001, *adj* ${\hat{\eta }}_{\text{p}}^{2}$ = 0.208. Pairwise comparisons revealed the same pattern of differences as was found for mRT. Neither the main effect of relevant dimension nor the interaction was reliable; both ***F*** < 1, *p* ≥ 0.611.

The second set of analyses concerned the effects of most-recent-pairing, ignoring correlation condition. In this case, there are only two possibilities: the current trial either required the same or the opposite response as the last trial involving the same value of the irrelevant attribute. For this analysis, not only did the immediately preceding trial need to be performed correctly (see above), but the most-recent trial involving the same irrelevant attribute also needed to be correct; otherwise, it would not be clear to which response the attribute was currently bound. The mRTs for same and opposite responses were 470.67 and 480.96 ms, respectively, with mERs of 3.0 and 4.4%. The main effect of most-recent-pairing on mRT was 10.29 ± 2.44 ms: ***F***(1, 30) = 17.30, *p* < 0.001, *adj* ${\hat{\eta }}_{\text{p}}^{2}$ = 0.345. The main effect on mER was 1.4 ± 0.4%: ***F***(1, 30) = 11.80, *p* = 0.002, *adj* ${\hat{\eta }}_{\text{p}}^{2}$ = 0.258. Neither the main effect of relevant dimension nor the interaction was reliable for either mRT or mER: all ***F***(1, 30) ≤ 1.25, *p* ≥ 0.273.

As a follow-up, the most-recent-pairing effect on mRT was further analyzed by dividing the data into two sub-sets as a function of the lag between the current and previous appearance of the irrelevant attribute. To approximately equalize the numbers of observations, short lag was defined as one to three trials; long lag was defined as four or more trials. In the ANOVA, neither the main effect of relevant dimension nor any interaction involving this factor was significant: all ***F***(1, 30) ≤ 2.40, *p* ≥ 0.132. In contrast, the interaction between most-recent-pairing and lag was reliable: ***F***(1, 30) = 5.70, *p* = 0.024, *adj* ${\hat{\eta }}_{\text{p}}^{2}$ = 0.132. Pairwise comparisons revealed a significant most-recent-pairing effect at short lags (18.43 ± 3.99 ms; *p* < 0.001), but not at long lags (4.66 ± 3.49; *p* = 0.191).

The last set of analyses re-examined the effect of correlational cuing while controlling for most-recent-pairing. This was done by omitting all trials for which the most-recent-pairing involved the same response—i.e., only opposite-response trials were retained—because same-response trials almost never occurred in the negative correlation condition and always involved an exact repetition of both stimulus attributes. To maximize the number of observations while still avoiding exact repetitions, both positive test trials and inducing trials were treated as positive correlation, because these two conditions have the same relationship between the irrelevant attribute and the correct response, and these two conditions produce near-equal performance (see, e.g., the first set of analyses, above). The mRTs for positive and negative correlation were 472.87 and 489.06 ms, respectively, with mERs of 3.5 and 5.1%. The correlational-cuing effect in mRT was 16.19 ± 3.78 ms: ***F***(1, 30) = 18.34, *p* < 0.001, *adj* ${\hat{\eta }}_{\text{p}}^{2}$ = 0.359. The same effect on mER was 1.7 ± 0.8%, but not reliable: ***F***(1, 30) = 4.12, *p* = 0.051. In neither analysis was the main effect of relevant dimension nor the interaction reliable: all ***F*** < 1, *p* ≥ 0.415.

One concern with this final analysis (raised by a reviewer) is the possibility that lag was confounded with correlation condition. Given that the effect of most-recent-pairing has been found to decrease with lag (see, e.g., Giesen et al., 2020; Schmidt et al., 2020; and the second analysis, above), this would be a serious problem, if true. On first look, it might seem that trials in the negative correlation condition would have higher lags, because these trials are quite rare (by definition). Alternatively, when one only retains the trials involving the opposite response (as was done for the final analysis), one might expect the lag to be higher for the positive condition, because the previous trial must have been negative. Fortunately, lag is only determined by the previous occurrence of the irrelevant attribute and this does not depend on correlation condition, even after controlling for most-recent-pairing. With six different values for the irrelevant attribute and trials being run in a pseudo-random order, the mean lag for all conditions will be approximately 6 (The actual mean lags for the positive and negative correlation conditions in the final analysis were 5.72 and 5.93, respectively). Even more, the distribution of specific lags should be the same for the positive and negative conditions, even after controlling for most-recent-pairing (This was verified by a χ^2^ test which produced a *p*-value of 0.683). In summary, the present finding of a significant effect of correlation condition while controlling for most-recent-pairing cannot be due differences in lag, as the lags were the same across the conditions.

### Discussion

In contrast to other recent work examining the effects of correlations between irrelevant stimulus attributes and available responses while controlling for instance-based confounds (Giesen et al., 2020; Schmidt et al., 2020), the present experiment was designed to de-emphasize repetitions by using a large number of different stimulus values and a many-to-one mapping between relevant stimulus values and responses. Under these conditions, we observed a significant correlational-cuing effect that remained reliable when most-recent-pairing was taken into account. This would appear to rule out the idea that temporary bindings are the entire source of correlational cuing. At the same time, the present work produced new evidence of most-recent-pairing effects, so this issue should not be ignored.

## Replication

One issue (raised by reviewers) that weakens the evidence that is provided by the experiment above is the mapping of “warm” vs. “cold” colors to different responses. This was done to equalize overall performance between the color-relevant and letter-relevant conditions, but opens the door to alternative explanations. Most of all: if the participants processed the colors as warm vs. cold, instead of as one of six different values, then the coding scheme that was used to analyze the data is flawed. To address this issue, we replicated the color-relevant condition of the experiment using a mapping that was designed to prevent participants from dichotomizing the colors as warm vs. cold. The new color sets were red, blue, and gold vs. green, orange, and purple. In other words, the assignment of colors to responses alternated, left/right, as one travels around the color circle. With respect to the example design provided by Table 4, the colors for the second (gold) and fifth (blue) rows were swapped. Thus, each set of colors included at least one warm value and at least one cold value. All other aspects of this replication of the color-relevant condition were exactly the same as above. A new sample of 16 participants was recruited.

The results from the replication were remarkably consistent with the previous experiment (other than a slight increase in overall mean RT, as expected). In the analysis of correlational cuing while ignoring most-recent-pairing, the effect of condition was significant: ***F***(2, 30) = 23.97, *p* < 0.001, *adj* ${\hat{\eta }}_{\text{p}}^{2}$ = 0.589. The mRTs for inducing, positive, and negative test trials were 494.37, 492.98, and 524.35 ms, respectively, and pairwise comparisons found significant differences between positive and negative test trials (31.37 ± 6.05 ms; *p* < 0.001) and between inducing and negative test trials (29.98 ± 5.00 ms; *p* < 0.001), but not between inducing and positive test trials (1.40 ± 4.13 ms; *p* = 0.982). As before, the effect of correlation condition on mER was also significant even after correction for a violation of sphericity: ***F***(1.41, 21.14) = 20.40, *p* < 0.001, *adj* ${\hat{\eta }}_{\text{p}}^{2}$ = 0.548. The mERs were 4.0, 3.8, and 8.9% for inducing, positive, and negative, respectively, and the pairwise comparisons matched those for mRT. Likewise, in the analysis of most-recent-pairing while ignoring correlation condition, the difference in mRT between same- (491.14 ms) and opposite-response trials (512.95 ms) was significant: ***F***(1, 15) = 19.31, *p* < 0.001, *adj* ${\hat{\eta }}_{\text{p}}^{2}$ = 0.534. The difference in mER (same: 4.0%; opposite: 6.4%) was also reliable: ***F***(1, 15) = 21.26, *p* < 0.001, *adj* ${\hat{\eta }}_{\text{p}}^{2}$ = 0.559. Finally, in the critical analysis of correlation cuing while controlling for most-recent-pairing, the mRT for positive and inducing trials (combined) was 501.48 ms and the mRT for negative trials was 524.35 ms. The effect of correlation condition was 22.87 ± 5.66 ms and significant: ***F***(1, 15) = 16.29, *p* < 0.001, *adj* ${\hat{\eta }}_{\text{p}}^{2}$ = 0.489. The mERs were 3.8 and 8.8% for positive and negative trials, respectively, which was also significant: ***F***(1, 15) = 16.45, *p* < 0.001, *adj* ${\hat{\eta }}_{\text{p}}^{2}$ = 0.491.

In summary, the replication was highly successful. The same pattern of results was found when the colors were mapped to responses in a manner that would prevent the participants from re-conceptualizing the task in terms of warm vs. cold, instead of six different colors. As before, the effect of correlation condition remained reliable when most-recent-pairing was taken into account. In fact, the marked similarity in the results between the two experiments suggests that the specific mapping of relevant values to responses is unimportant. However, it’s worth noting that these two experiments have only used a small number of the myriad ways in which six different irrelevant values can be correlated with two different responses. It remains possible that the specific assignment of irrelevant values to correlation condition plays some role and this should be addressed in future research, possibly by employing (more) complete counter-balancing. It would also be useful to extend these findings to other stimulus sets, such as replacing the letters with words.

## General Discussion

In both real-world and laboratory situations, supposedly irrelevant attributes of stimuli are often correlated with or predictive of the appropriate action or correct response. When this is true, one observes a correlational-cuing effect: an advantage for trials on which the correlation is positive, rather than negative. One possible explanation for this finding is that people learn and use the correlations via the same mechanisms as proposed by classical learning theory (e.g., Pavlov, 1927; Rescorla and Wagner, 1972). This process depends on all previous experiences with the stimuli and responses, combined by a process of association updating (e.g., the delta rule). An alternative explanation is that these effects are due to the transient binding of features (e.g., Hommel, 1998, 2004). This process depends on exactly one previous experience: the most-recent event that involved the same value of the irrelevant attribute.

Until quite recently, the modal view of correlational cuing in the context of simple tasks has been that it parallels classical learning (see, e.g., Miller, 1987; Danek, 2010; Schmidt and De Houwer, 2016a,b). A serious challenge to this idea was recently provided by two studies that found no significant contribution of correlation condition when recent events were taken into account (Giesen et al., 2020; Schmidt et al., 2020). It could be argued, however, that these studies were not optimized for the associative learning of correlations. The present experiment employed conditions that were designed to be much more favorable to classical learning and found significant correlational cuing even when the most-recent-pairings were matched.

In addition to the present approach, there are at least two other lines of argument against models that only involve most-recent-pairings or limit the retrieved episodes to those that are recent. First, several studies have found persistent effects of correlation condition even after the correlations have been removed (Danek, 2010; Cosman et al., 2016; Schmidt and De Houwer, 2016b; Lin and MacLeod, 2018). When the correlations are removed, the differences in transient bindings or retrieved recent episodes would no longer support the effect, but the effect is still observed, often for several blocks of trials. In contrast, models based on associative learning are entirely consistent with these results, because extinction always takes time and the earliest experiences often have the largest effects due to primacy (see, e.g., Pineño and Miller, 2005). Second, the very first paper on this general topic included an experiment in which letters that had previously only been correlated with certain responses became the actual targets for a new task (Miller, 1987, Expt. 3). Mapping the letters onto the same responses with which they had previously been correlated provided a significant performance advantage over mappings that reversed the relationships. Binding- and retrieval-based models have some difficulty explaining this finding, because the tasks were quite different and no repetitions of specific displays were involved.

It should be noted, however, that the binding-based model tested here is just one of the possible instance-based theories. Other models that propose that many or all previous experiences are involved remain viable. What is needed is a method of discriminating between models based on all previous experiences *combined* (consistent with associative learning) and those based on all previous experiences *separately* (as under Parallel Episodic Processing; Schmidt et al., 2016). One possible approach to this question would employ computational modeling, as this proved quite useful when a similar question arose in the context of automaticity (see, e.g., Logan, 1988). Some models of automatization and habit formation involve associative learning; other models of the same are based on the retrieval of separate instances (for an extensive discussion, see Giesen et al., 2020).

While not parsimonious, at this point, the safest interim conclusion is that both associative learning and instance-based mechanisms exist, and that each plays a role, with their relative contributions depending on specific conditions. In this regard, three factors that should be explored in future work are task complexity (e.g., one-to-one vs. many-to-one mappings of stimuli to responses), the number of different values for each attribute (or some other method of varying the frequency of stimulus repetitions), and the overall strength of the correlation. It would useful to know when and why each of these distinct mechanisms—associative learning, transient binding, and episodic retrieval—is active.

## Data Availability Statement

The raw data supporting the conclusions of this article will be made available by the authors, without undue reservation, to any qualified researcher.

## Ethics Statement

The studies involving human participants were reviewed and approved by the Institutional Review Board, University of Iowa. The patients/participants provided their written informed consent to participate in this study.

## Author Contributions

GX and JM contributed equally to all aspects of this research. Both authors contributed to the article and approved the submitted version.

## Conflict of Interest

The authors declare that the research was conducted in the absence of any commercial or financial relationships that could be construed as a potential conflict of interest.
